# Hampering Stromal Cells in the Tumor Microenvironment as a Therapeutic Strategy to Destem Cancer Stem Cells

**DOI:** 10.3390/cancers13133191

**Published:** 2021-06-25

**Authors:** Katherine Po Sin Chung, Rainbow Wing Hei Leung, Terence Kin Wah Lee

**Affiliations:** 1 Department of Applied Biology and Chemical Technology, The Hong Kong Polytechnic University, Hong Kong, China; katherines.chung@connect.polyu.hk (K.P.S.C.); wing-hei-rainbow.leung@polyu.edu.hk (R.W.H.L.); 2 State Key Laboratory of Chemical Biology and Drug Discovery, The Hong Kong Polytechnic University, Hong Kong, China

**Keywords:** cancer, cancer stem cells, immune cells, stromal cells, tumor microenvironment

## Abstract

**Simple Summary:**

Cancer stem cells (CSCs) are regarded as the root of tumor development and drug resistance. Accumulating evidence shows that the behaviors of CSCs are highly regulated by stromal cells within the tumor microenvironment (TME), and concurrently CSCs regulate the function of various immune cells. In this review, we focus on the crosstalk between stroma cells and cancer cells, which leads to CSC expansion, drug resistance and immune evasion. Understanding the role of stromal cells in regulation of the above processes reveals novel potential therapeutic strategy against cancer.

**Abstract:**

Cancer stem cells (CSCs) within the tumor bulk play crucial roles in tumor initiation, recurrence and therapeutic resistance. In addition to intrinsic regulation, a growing body of evidence suggests that the phenotypes of CSCs are also regulated extrinsically by stromal cells in the tumor microenvironment (TME). Here, we discuss the current knowledge of the interplay between stromal cells and cancer cells with a special focus on how stromal cells drive the stemness of cancer cells and immune evasive mechanisms of CSCs. Knowledge gained from the interaction between CSCs and stromal cells will provide a mechanistic basis for the development of novel therapeutic strategies for the treatment of cancers.

## 1. Introduction of the Relationship between CSCs and TME Components

Accumulating evidence suggests the involvement of cancer stem cells (CSCs) in the perpetuation of various cancers, including acute myeloid leukemia [1], brain [2], colon [3], breast [4], liver [5] and prostate cancers [6]. CSCs are regarded as the root of tumor initiation, progression and therapeutic resistance [7]. The development and progression of cancer have long been considered cell-autonomous processes in which progressive genetic and epigenetic alterations transform cells without regard for the external context. In fact, cancers are composed of not only tumor cells but also various types of stromal cells within the tumor microenvironment (TME), including fibroblasts, immune cells and endothelial cells. Similar to normal stem cells, CSCs are regulated by both intrinsic [8] and extrinsic signals [9], which are generated by the TME in the case of CSCs. Therefore, a better understanding of how the properties of CSCs are regulated by various cellular factors could lead to the development of a novel therapeutic strategy for targeting CSCs. In this review, we summarize the tumor-sustaining crosstalk between cancer cells and stromal cells, encompassing endothelial cells, adipocytes, myeloid-derived suppressor cells, tumor-associated neutrophils, regulatory T cells, tumor-associated macrophages and cancer-associated fibroblasts that drive the stemness of CSCs. There is growing evidence that immune cells not only drive CSC expansion but also elicit CSC-specific avoidance of immune detection and destruction. Finally, we also explore novel therapeutic targets crucial for the crosstalk between stromal cells and cancer cells in the hope of developing a new avenue for cancer therapy.

## 2. The Interplay between Stromal Cells and CSCs within the TME

Accumulating evidence has shown the interrelationship between CSCs and cellular factors within the TME. Herein, we begin by first reviewing the current knowledge of the interactions between CSCs and various stromal cells, including endothelial cells, cancer-associated fibroblasts and adipocytes, that lead to the regulation of CSC plasticity via recruitment and expansion of these cells. Understanding the interactions between CSCs and stromal cells enables us to develop a therapeutic strategy to destem CSCs.

### 2.1. Endothelial Cells

Angiogenesis is critical to maintain the supply of nutrients and oxygen required to support tumor growth. Endothelial cells, especially vascular endothelial cells that line blood vessels, play a major role in maintaining CSCs and facilitating tumor metastasis. In colorectal cancer spheroids, the CSC population expands, and the expression of the stem cell markers NANOG and OCT4 increases when cultured with conditioned medium from endothelial cells of noncancerous organs, including the liver, lung, colon mucosa and kidney [10]. Previous studies have revealed that soluble factors secreted by endothelial cells are able to maintain stem cell properties in neural stem cells; therefore, secretions from endothelial cells could also activate CSCs and promote tumor growth [11]. In a coculture setup, Nestin^+^CD133^+^ brain CSCs and endothelial cells were shown to selectively interact in the tumor niche. Endothelial cell expansion and vascularization promote tumor initiation and the self-renewal ability of CSCs through the release of the endothelial cell-derived secretome [12]. A study by McCoy et al. revealed that perivascular endothelial cells secrete IL-8 and enhance the CSC characteristics of glioblastoma cells, including their migration and invasion abilities [13]. Moreover, glioblastoma tumor cells induce endothelial cell migration toward the tumor bulk. With this positive feedback loop of IL-8-mediated signaling, endothelial cells promote brain tumor growth. Fessler and his team also reported that endothelial cells can induce CSC properties in differentiated glioblastoma cells. With bFGF supplementation, CD133^−^O4^+^ cells were capable of regaining CD133 expression, exhibiting an increase in CSC properties [14]. EGF, another growth factor secreted by endothelial cells, also exhibits the potential to induce the CSC phenotypes of metastasis by epithelial-mesenchymal transition (EMT) and drug resistance in squamous cell carcinoma [15]. Overexpression of epiregulin (EREG) in esophageal cancer induced by endothelial cells demonstrated an increase in actin rearrangement, spheroid formation and enrichment of CD44^+^ CSCs [16]. In addition to endothelial cell-mediated promotion of angiogenesis, endothelial cell secretions, mainly growth factors, enhance CSC phenotypes. Interestingly, approximately two-thirds of the endothelial cells in brain tumors carry a portion of genomic mutations identical to those in brain CSCs. The endothelial lining of the vasculature is comprised of a small population of cells with tumor-initiating properties, showing their neoplastic origin [17]. This finding indicates that the interactions between endothelial cells and CSCs are not simple and unidirectional but are reciprocal. A portion of CSCs can even differentiate into endothelial cells in the TME to support and sustain tumor growth. To target the tumor-promoting effect of endothelial cells, an IL-8 blocking antibody was applied in a mouse model established via the intracranial injection of tumor cells or a blend of tumor cells and endothelial cells. IL-8 suppression by the antibody resulted in a reduction in tumor size. This phenomenon was exclusively observed in the tumor-endothelial cell mixture but not in mice injected with tumor cells only [13]. Therefore, neutralization of endothelial cell-derived IL-8 production could be a potential therapy for cancers, including glioblastoma.

### 2.2. Cancer Associated Fibroblasts

Cancer-associated fibroblasts (CAFs) are a dominant stromal cell type in the TME that are activated from resting fibroblasts via the NF-κB and JAK-STAT pathways once cancer cells or immune cells release signaling molecules such as TGFβ, RTK ligands, IL1β and IL6 [18]. Thus, CAFs can gain the ability to produce the extracellular matrix (ECM) and molecules essential to maintaining tumor growth and the properties of CSCs, further promoting therapeutic drug resistance [19,20]. A recent study showed that there is a positive feedback loop between CSCs and CAFs in the niche that supports cancer cell stemness in various cancers [21]. Several studies have also revealed that the factors secreted by CAFs induce EMT, which can further enhance the properties of CSCs [22,23]. Induced EMT causes cisplatin resistance in non-small-cell lung carcinomas (NSCLCs) [24], and CSC properties can be sustained with chemoresistance in both breast cancers and NSCLCs [25]. Therefore, inactivating CAFs or their corresponding activating molecules to lower the level of infiltrating CAFs in the TME are potential therapeutic strategies for reducing cancer stemness [26]. For example, targeting myofibroblast-like CAFs by FAK inhibitor resulted in a reduction of CSCs in pancreatic cancers [27], whereas blocking the paracrine signaling of IGF-II/IGF1R/Akt/Nanog lowered the density of CSCs in lung cancers [28]. In breast cancers, Pelon et al. classified four types of CAFs, two of which are involved in invading cancer cells [29]. CAF-S1 enhances migrating cells and EMT through the secretion of TGFβ, while CAF-S4 increases invading cells [29]. In addition, hindering the activation of CAFs sensitized CSCs to chemotherapeutic treatment [30]. Studies have also shown that CD44 expression on CAFs is a functional target for destroying CSCs in the TME both in vitro and in vivo [31], and TGFβ signaling mediated by CAFs plays a role in regulating CSCs in gastric cancers [32]. We have also shown that targeting the c-Met/FRA1/HEY1 cascade mediated by HGF could be a promising treatment strategy for hepatocellular carcinoma (HCC) since the tumor-initiating cells of the liver are regulated by CAFs together with HGF secretions [33]. Targeting CAFs also affects other stromal cells, such as polarizing TAMs and suppressing the cytotoxic activities of NK cells, since CAFs are involved in promoting immunosuppression [18]. Therefore, hindering CAFs not only reduces M2-type TAMs and destroys the CSC niche but also increases NK cell functions, which will be discussed in later sections. These findings suggest that disabling the crosstalk between CAFs and CSCs is a convincing strategy for reducing drug resistance, metastasis and the stemness of CSCs. However, there is also some evidence indicating that depleting CAFs is not always beneficial, as it has been shown to promote angiogenesis and enhance CSC properties in pancreatic cancer, with shorter patient survival [34]. Further preclinical studies have shown that deleting stromal fibroblasts may inhibit the control of tumor growth [35]. Sahai et al. showed that the vitamin D receptor can act as a target after confirming the subtypes of CAFs or reprogramming of CAFs, which potentially hinders the progression of pancreatic cancer cells [36]. In breast cancers, there are some novel therapeutic strategies to target CSC-CAF interactions. GW4064 is an agonist of farnesoid X receptor (FXR), which helps decrease the signaling of leptin [37,38], while pirfenidone and doxorubicin are drugs that can inhibit the production of collagen [39]. When these two drugs are combined, the progression and motility of tumors are reduced, with changes in ECM components.

### 2.3. Adipocytes

Recently, increasing evidence has suggested the existence of an obesity/cancer axis due to the positive correlation between adipose tissue and multiple cancers. Adipocytes, as the predominant cells in adipose tissue, have been shown to sustain CSC properties through paracrine secretion to the TME [40]. Interestingly, adipocytes participating in cancers are referred to as cancer-associated adipocytes, as they exhibit different phenotypes and effects on cancers compared to normal adipocytes. By coculturing adipocytes with cancer cells, a surge in the levels of different proteases and cytokines (such as IL-6 and IL-1β) and a reduction in adipocyte-related markers are observed [41]. Leptin is the primary adipokine secreted by adipocytes and activates the proliferation and migration of tumor cells. With the elevated level of leptin, surrounding adipocytes in the bone marrow microenvironment support the proliferation and migration of multiple myeloma cell lines and protect them against apoptosis by suppressing caspase-3 activity [42]. Leptin signaling enriches breast CSCs by increasing receptor expression levels and activating Notch and Wnt stem cell pathways [43] as well as oncogenic HER2, AKT and NF-κB pathways to promote tumor formation and invasion [44]. Other studies on the interaction between breast cancer and adipocytes have revealed that the inflammatory factor IL-6 is the other key player in maintaining cancer stemness. In a coculture milieu of breast cancer cell lines and adipocytes, the expression and secretion of IL-6 leads to an increase in the metastatic potential of cancer cells by the upregulation of PLOD2 expression [45]. Enrichment of IL-6 by adipocytes in the TME preferentially regulates Bcl_xl_ expression in CD44^+^/MyD88^+^ epithelial ovarian CSCs, contributing to chemoresistance [46]. Secreted IL-6 enhances the expression of OCT4 through the regulation of STAT3. When compared with the differentiated cells of triple-negative breast cancer cells, the stemness-bearing CD44^+^CD24^−^ cell population has a preferentially higher activity in the IL-6/JAK/STAT3 pathway, leading to CSC proliferation and tumor growth [47]. The expression of an additional extrinsic factor, obesity-associated fatty acid binding protein 4 (FABP4), is elevated in patients with breast cancer. FABP4 supplementation increases tumor volume, tumor-initiating frequency and stemness markers, as shown in in vivo and in vitro studies on mammary tumors, depending on the IL-6/STAT3/ALDH1 signaling pathway [48]. Adipocyte-associated secretion of IL-6 also participates in the Notch/Wnt/TGF-β signaling pathways by upregulating ALDH1A1 [49] and LEF1 and AXIN2 gene expression in the Wnt pathway [50] to enhance the invasiveness, metastasis and angiogenesis of breast cancer. To target the interaction between adipocytes and cancer cells, BMS309403, a FABP4-specific inhibitor, was evaluated in an orthotopic breast cancer mouse model. Hao et al. demonstrated a significant reduction in tumor growth with changes in both IL-6 secretion and ALDH1 expression [48]. Furthermore, another study showed that an anti-leptin blocking peptide abrogates the migration ability of ovarian cancer cells [51]. To improve the purity and reduce the endotoxicity of this peptide, antibodies against leptin should be developed for effective cancer therapy.

## 3. The Interplay between Immune Cells and CSCs within the TME

Recent studies have begun to elucidate the relationship of CSCs with immune cells. The interaction between cancer cells and immune cells is reciprocal. Apart from the role of particular immune cell types in driving CSC expansion, increasing evidence has also demonstrated the distinct ability of CSCs to evade surveillance and destruction by immune cells [52]. Understanding the molecular mechanism of how CSCs evade the immune system may help to identify strategies to eradicate the subpopulations of cancer cells that escape elimination by conventional therapy. Herein, we review the current knowledge of the interactions between CSCs and immune cells that lead to the regulation of CSC plasticity and immune evasion and explore the interactions between CSCs and tumor immunology.

### 3.1. Tumor Associated Macrophages

Tumor-associated macrophages (TAMs) are classified into two groups—tumor-suppressing M1-TAMs and tumor-promoting M2-TAMs—that infiltrate the tumor microenvironment and promote tumorigenicity [53]. The majority of clinical studies have shown that increasing numbers of TAMs lead to poor survival in patients with cancer, including breast cancer, lung cancer, thyroid cancer and HCC [54,55]. With protumoral abilities, angiogenesis can be induced, adaptive immunity can be suppressed, and the extracellular matrix can be remodeled [56]. Although there are still some limitations in disturbing the crosstalk between TAMs and CSCs, either reducing the accumulation of TAMs or reprogramming them into antitumor macrophages can lower the burdens and metastatic abilities of tumors [57]. Depleting the tumor-derived molecules that assist in promoting the recruitment of monocytes is important to suppressing the accumulation of TAMs [58]. CCL2 and CSF-1 levels are high in multiple cancers and are directly proportional to the density of monocytes recruited [59]. There is also in vivo evidence suggesting that the migration of macrophages to tumors and subsequent tumor invasion are enhanced when the matrix is disrupted with either TAM-derived EGF or CSF-1 and inhibiting either of these molecules results in the opposite effect [60]. For example, downregulation of CCL2 reduces tumor growth in prostate cancers [61] and inhibits cancer stem cell properties in breast cancers [62], and targeting CSF-1 can reduce TAMs and enhance the ratio of CD8^+^ to CD4^+^ T-cells [63]. Apart from targeting TAMs, reprogramming M2 phenotypes showed promising results for inhibiting CSC phenotypes [58]. When M2 TAMs were reprogrammed, the production of IL12 was blocked, leading to inactivation of antitumor responses [64]. However, accumulation of M2-TAMs promoted the polarization of Th2 cells and further induced IL-4, leading to an even greater M2-TAM population [65]. Studies have suggested that inhibiting the signaling of TGFβ with TLR7 ligation helps reprogram the phenotype of TAMs [66], whereas the blockade of TGFβ signaling can reduce the progression of tumors and liver CSC properties [67,68]. Furthermore, the β-catenin pathway plays a crucial role in tumor development by regulating the reprogramming of M2-type TAMs. For instance, in lung cancers, Wnt/β-catenin signaling involved in M2/M1 transitions suppresses the growth of tumors [69]. Additionally, since the secretion of certain cytokines by the activated STAT3 pathway could enhance the recruitment of monocytes and M2-type TAMs, CAFs can also be activated and thus progress to tumor cells and promote invading cells, as proven in squamous cell carcinomas [70,71]. Therefore, there is an interrelationship between TAMs and CAFs, and targeting the recruitment of monocytes/macrophages and repolarizing the M2 phenotype to the M1 phenotype can reduce the stemness of CSCs in tumors through the destruction of the supportive CSC niche. There are some novel therapeutic strategies to target TAMs available. For example, CD40 agonists help activate and induce the functions of M1-type macrophages, which results in ECM degradation and redirects inflammatory monocytes/macrophages to induce fibrosis degradation and thus suppress tumor outgrowth [72]. However, an IL-33 neutralizing antibody helps block IL-33 in NSCLCs. As a result, it inhibits the polarization of M2-type macrophages by hampering IL-10 and VEGF secretion, which in turn reduces the accumulation of Treg cells [73].

### 3.2. Natural Killer Cells

Natural killer cells (NK cells) can be classified into two types, with the ability to kill cancer cells depending on the balance between the expression of activating (mostly stress-induced proteins) and inhibitory (in particular MHC class I molecules) ligands on the surface of target cells. Over 90% of NK cells are CD56^dim^CD16^bright^, while approximately 5% of them are CD56^bright^CD16^dim^. Stronger cytotoxicity can be exerted by CD56^dim^CD16^bright^, whereas the cytotoxic activity of CD56^bright^CD16^dim^ is mediated via the production of cytokines [74]. Apart from these two classical types, there are some NK cells marked by CD56^dim^CD16^dim^, which play a tumor-promoting role by augmenting angiogenesis [75]. Clinically, these NK cells are found at the tumor site of leukemia patients [76]. Other types of NK cells called decidual NK (dNK) cells, which are marked by CD56^superbright^CD16^dim^, were also reported to play a tumor supportive role via a similar mechanism [77]. Specifically, dNK cells activate angiogenesis by releasing angiogenic factors such as VEGF and angiogenin [78]. Keskin et al. discovered that TGFβ converted CD16^+^ to CD16^−^ NK cells, and these cells functionally behave similarly to decidual NK cells in NSCLC patients [79]. Furthermore, Antonino and his team also reported that the other subtypes that are dNK-like are the tumor-associated NK (TANK) cells and tumor-infiltrating NK (TINK) cells, as they have similar proangiogenic features [80]. TANKs promote both MMP2/9 (matrix metalloproteinase 2/9) and TIMP (tissue inhibitors of metalloproteinase) through the activation of STAT3/STAT5 in colorectal cancer. As a result, these kinds of NK cells induce invasion and metastasis of colorectal cancer cells [81]. From the above data, we believe that NK cells possibly regulate cancer stemness via upregulation of VEGF and STAT3/5. Therefore, targeting certain subtypes of NK cells may help reduce the angiogenic ability of CSCs.

Recently, CSCs have been found to evade the attack of NK cells. Cheung et al. showed that liver CSCs marked by granulin-epithelin precursor (GEP) suppressed NK activation via production of soluble MICA, which could be reversed by antibody blockade against GEP [82]. Therefore, targeting CSCs with NK cells is one of the promising approaches for CSC-targeted immunotherapy. NK cells can recognize surface natural cytotoxicity receptor (NCR) ligands, including activating and inhibitory receptors, on target cells and thus lyse CSCs [83,84]. Studies have shown that NK cells attack CSCs by activating receptors that are expressed upon viral infection and cell proliferation [85], and the survival and cytotoxic activity of NK cells can be enhanced by the activation of either IL2 or IL15 [86]. Therefore, activating NK cells could be a possible way to eliminate CSCs. For instance, both CSCs and tumor burdens were significantly reduced with NK-activating cells in pancreatic cancers [87], and higher NK-activating ligands with lower NK-inhibitory ligand expression resulted in preferential recognition and killing of colorectal CSCs [88]. In malignant gliomas, especially glioblastoma, TGFβ is responsible for regulating NKG2D expression, and inhibiting TGFβ not only causes better recognition and lysis by NK cells with an activated phenotype but also leads to a comparatively lower migration and invasion ability [89]. Furthermore, IL2-activated NK cells successfully kill melanoma CSCs, which was proven to be a novel method to target melanoma metastasis in the past [90]. Ultimately, there is still a need to investigate how to reduce CSCs by targeting NK cells, but it is understood that CSCs can be eliminated through recognition and lysis by NK cells. As therapeutic treatments for both solid and liquid tumors, chimeric antigen receptor-engineered natural killer (CAR-NK) cells can be used, which potentially recognize and target corresponding antigens preferentially expressed in CSCs and specifically eliminate them [91].

### 3.3. Myeloid-Derived Suppressor Cells

Myeloid-derived suppressor cells (MDSCs) are a group of immune cells in the tumor bulk characterized by high levels of iNOS and arginase that maintain their suppressive effect on T cell activity [92,93]. MDSCs are suggested to be heterogeneous and originate from myeloid cells under states of chronic inflammation, cancers and infections [94]. A recent study showed that MDSCs produce PGE2 to strengthen the stemness and PD-L1 expression of ALDH^High^ ovarian CSCs via the activation of the PI3K/AKT/mTOR signaling pathway [95]. Elevated PD-L1 expression is suggested to be linked with PD-L1-dependent suppression of T cells, leading to tumor growth and metastasis [96]. Peng et al. [97] reported the coordinative role of CD33^+^ MDSCs in immune suppression and escape through the enhancement of breast cancer CSC properties. This population of MDSCs promotes self-renewal and stimulates the expression of stem cell markers in CSCs. With the release of IL-6 and nitric oxide, phosphorylation of STAT3 and activation of the NOTCH signaling pathway, the crosstalk effects between MDSCs and breast cancer cells are activated. This feedback loop system is reinforced by both IL-6/STAT3 and NO/NOTCH, causing sustained activation of STAT3 [97]. The study of MDSCs in pancreatic cancer has also shown that STAT3 activation is the mediator for enhancing the stemness of CD24^+^, CD44^+^ and ALHD1^Bright^ CSC populations and promoting metastasis in a mouse model [98]. In recent decades, immunostimulatory RNA molecules have been found to participate in various cancers through crosstalk with MDSCs [99,100]. Cui et al. suggested that MDSCs in ovarian cancer suppress T cell functioning and enhance CSC renewal and cancer metastasis, which are primarily triggered by miRNA101 through the inhibition of CtBP2 and targeting of stem cell genes [101]. In multiple myeloma, high granulocytic MDSCs are correlated with poor overall survival. This subset of MDSCs with elevated expression of core stem cell genes leads to drug resistance and tumor recurrence, as proven by a human xenograft model. This study also revealed that the stemness of multiple myeloma is maintained by piRNA-823 and DNMT3B through crosstalk with MDSCs [102]. The mechanism by which MDSCs regulate CSC stemness through RNA molecules remains unclear. However, one feasible mechanism is the transfer of RNA molecules via exosomes, which are small double-membrane vesicles that transport various components into recipient cells, aid intercellular communication, and, in particular, play a role in cancer metastasis [103,104]. In endometrial cancer, targeting MDSCs with a Gr-1 neutralizing antibody showed a decrease in MDSC populations. Despite the fact that attenuation of MDSC function by celecoxib showed only a limited inhibitory effect on tumor growth, both strategies in combination with doxorubicin were suggested to reduce ALHD^+^ expression and sensitize tumor cells to chemotherapy, leading to tumor suppression [105].

### 3.4. Tumor-Associated Neutrophils

Neutrophils are often associated with the presence of MDSCs in chronic inflammation. Neutrophils also originate from myeloid precursors and are responsible for the innate immune response, and their functions in cancers can be polarized to antitumor N1 phenotypes or protumor N2 phenotypes [106]. Recent studies on tumor-associated neutrophils (TANs) have shown the plasticity of their procancer role in different malignant diseases [107]. In a study of TANs in HCC by Zhou et al., the release of chemokines CCL2 and CCL17 by TANs was increased relative to peripheral blood neutrophils. These chemokines attract macrophages and regulatory T cells, leading to tumor growth progression and resistance to targeted therapies in combination with the activation of the AKT signaling pathway [108]. Growing evidence suggests that TANs are associated with cancer metastasis. CXCR2^−^dependent recruitment of neutrophils through TNF-α activation in breast cancer and the angiogenic factor MMP-9, specifically delivered by TANs in lung cancer, are also evidence of the metastatic role of TANs [109,110]. TANs have been shown to support the proliferation of CD24^+^CD90^+^ breast CSCs and tumorigenesis via the MAPK and ERK pathways. Another study in which CXCR2 was blocked to suppress neutrophil migration also revealed a reduction in angiogenesis and tumor growth in melanoma [111]. Reciprocally, CSCs also exhibit a neutrophil-attracting function through paracrine secretion. Metastatic melanoma cells with elevated IL-8 expression cause infiltration of TANs, stimulating migration and invasion of metastatic cells to the lungs [112]. In addition, a positive feedback loop is achieved by TANs facilitating EMT in breast cancer through TIMP-1 secretion, while CD90^+^ breast cancer cells that undergo EMT in turn reinforce TAN infiltration through cell-cell interactions [113]. Moreover, CD133, a well-established CSC marker, has been shown to exert functional effects in the TME. The CD133 molecule in glioma cells aids neutrophil migration with the assistance of IL-1β, CCL3 and CXCL, causing tumor growth and resistance to anticancer therapies [114]. Wculek and Malanchi reported that elevated levels of neutrophil-derived leukotrienes were detected in the TME. These leukotrienes were characterized as Alox5 enzymatic products, which lead to expansion of CD24^+^CD90^+^ cells with high metastatic potential. By targeting Alox5 with zileuton (an Alox5 inhibitor), the spontaneous metastatic rate and the seeding capacity of cancer cells into the lungs were greatly reduced in a metastatic breast cancer mouse model [115].

### 3.5. Regulatory T Cells

T cells are a group of heterogeneous immune cells, and the regulatory T cell (Treg) subset is characterized by the expression of CD4^+^, FOXP3^+^ and CD25^+^ [116]. Previous reports suggest that the regulatory function of Treg cells suppresses their antitumor effect, which is related to poor survival and promotion of cancer stemness [117,118]. The presence of CSCs is associated with a high level of Treg cells, while the population of Treg cells is increased in parallel with the CSC population during cancer progression, showing their tumor-promoting effect [119,120]. Xu et al. indicated that breast cancer cells with Sox2 upregulation recruit Treg cells to the TME by the secretion of CCL1. Treg cells promote ALDH^bright^ expression and self-renewal ability in several breast cancer cell lines, leading to enhanced tumor initiation, invasiveness and chemoresistance [121]. Infiltrating Treg cells in glioma upregulate the expression of the core stem cell markers CD133, SOX2 and NESTIN in glioma stem cells, which are enriched by TGF-β secretion. TGF-β induces the release of IL-6 via the NF-κB signaling pathway, whereas IL-6 promotes cancer stemness through STAT3 activation [122]. Activation of TGF-β induced by Treg cells also enhances EMT and a subsequent increase in the migration and invasion of melanoma [123]. Under hypoxic conditions, a subset of Tregs release IL-17 and cause a significant increase in the expression of the stem cell markers CD133, CD44 and EpCAM. Using a coculture assay, the promotion of stemness in colorectal cancers induced by Treg cells was suggested to be mediated through both the AKT and MAPK signaling pathways [124]. Furthermore, the high Treg population in the bone marrow of acute myeloid leukemia (AML) patients parallels the leukemic stem cell percentage. After culturing with conditioned medium from Treg cells, the side population and self-renewal ability of AML cells were enriched, showing an increase in CSC stemness. These suppressive effects of Tregs on AML are mediated by IL-10 through the PI3K/AKT signaling pathway [125]. Concomitantly, angiogenesis and the vascular niche are also crucial for Treg cells to indirectly modulate cancer stemness. The pivotal player in angiogenesis, VEGF, has been revealed to promote CSC properties, and its level is related to the recruitment of Treg cells [12,126]. Depletion of Treg-induced cytokines may shed light on some feasible approaches for cancer treatment. Tocilizumab is an FDA-approved drug in humans targeting the IL-6 receptor that is widely used in rheumatoid arthritis treatment. Liu et al. explored a new application of tocilizumab in glioma. Through injection of tocilizumab in glioma xenografts, tumor progression was greatly retarded, and Treg-induced cancer stemness was abolished [122]. Another approach is to block the function of IL-10 with an IL-10R neutralizing antibody. IL-10 blockade suppressed Treg-induced sphere formation ability and the expression of stem cell markers, including OCT4 and NANOG [125]. Direct cell surface targeting of Tregs is also a possible therapeutic approach for cancer treatment. Targeting Tregs with a CD25 antibody resulted in a reduction in VEGF-induced tumor vascularization, while blockade of VEGFR2 decreased the CSC properties of cancer cells [126,127].

## 4. Interplay of Various Cellular Factors within the TME in the Regulation of Cancer Stemness and Immune Evasion

In the previous sections, we discussed how individual stromal cells and immune cells interact with CSCs within the TME. However, stromal cells do not regulate the plasticity of CSCs in isolation and have a highly context-dependent mechanism of action. As one of the major stromal cells in the TME, CAFs interact with various stromal cells and immune cells in the TME to regulate CSC plasticity and immune evasion. First, a number of reports have demonstrated the crosstalk between CAFs and macrophages in the promotion of cancer stemness. CAFs play a crucial role in the promotion of cancer stemness in HCC by reciprocally inducing the activity of TAMs [128]. Overexpression of these markers, including α-SMA and CD68^+^^,^ is associated with HCC recurrence and shorter overall survival [128]. Recently, Yang et al. showed that CAFs express endosialin, which regulates macrophage recruitment and polarization to support HCC progression [129]. In prostate cancer (PCa), Comito et al. identified crosstalk among different cellular components, including CAFs, TAMs and PCa cells, leading to the promotion of cancer stemness [130]. Apart from TAMs, CAFs also interact with endothelial cells in the TME. CAFs regulate the endothelial lipoma-preferred partner (LPP) gene in endothelial cells, rendering a chemoresistant phenotype in ovarian cancer [131]. Moreover, Song et al. reported novel cytokine-mediated crosstalk among CAFs, HCC cells and TANs, augmenting cancer stemness and TAN recruitment in HCC [132]. Specifically, CAF-derived CLCF1 recruited and promoted N2 polarization of TANs via induced secretion of CXCL6 and TGF-β in HCC cells [132]. Accumulating evidence has also demonstrated the interaction between CAFs and immune cells in promoting an immunosuppressive environment. CAFs promoted recruitment of CCR2+ monocytes and conversion to the MDSC phenotype via preferential secretion of CCL2, which created an immunosuppressive environment [133]. Targeting the CAF-MDSC axis by CCR2 inhibition may open a promising therapeutic avenue for converting from a non-T-cell-inflamed TME to a T-cell-inflamed counterpart in lung carcinoma [133]. In addition to MDSCs, the CAF-neutrophil axis was reported to be a promising approach for the development of stromal treatments in pancreatic cancer. CAF-derived CXCL12 recruited neutrophil infiltration, which led to resistance to T-cell mediated killing [134]. In addition, CAFs recruit and enrich the Treg population via IL6 secretion in esophageal cancer [135]. Last, CD73^+^ CAFs suppressed T cell activity in a colon cancer model via A_2A-_mediated immune suppression, and thus targeting the CD73-adenosine pathway is a promising approach for complementing PD1 therapy [136].

## 5. Conclusions

**Figure 1:**
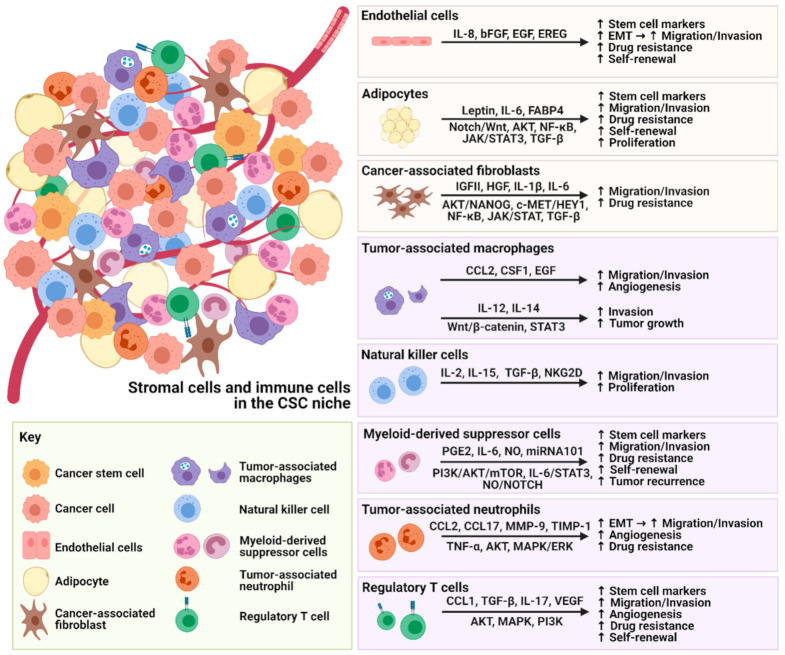
Illustration of the crosstalk between stromal cells, immune cells and CSCs.

The dynamic interactions between stromal cells and CSCs are not simply unidirectional but reciprocal, promoting the expansion of stem cell markers, migration, invasion, drug resistance and self-renewal properties of CSCs (Figure 1).

Similar tumor-promoting crosstalk is also observed with immune cells, leading to immune evasion of CSCs and tumor recurrence. An increasing number of studies have focused on targeting the major secretomes or molecules related to stromal cells and immune cells in the TME, which are summarized in Table 1. These therapeutic inhibitors or neutralizing antibodies could potentially be used as single treatments or in combination with current therapies for better treatment outcomes. However, it is not yet clear whether these promising preclinical strategies that enhance our understanding of cancer development will translate into effective treatment for cancers. More knowledge needs to be gained, and convincing preclinical results need to be evaluated in clinical trials—there is still a long way to go. Although these targets have been reported, their effects on cancer phenotypes may be tumor-type specific. Some solid tumors and liquid tumors may have different responses to stromal cells and immunotherapy. Therefore, hampering stromal cells as a therapeutic strategy to destem CSCs needs further investigation.

## 6. Future Perspectives on CSC-Targeted Therapies

Targeting stromal cells has become one of the strategies to destem CSCs. Many preclinical and clinical trials are being conducted to evaluate the therapeutic efficacies of various small molecule inhibitors/neutralizing antibodies in disrupting the interactions between stromal cells and cancer cells. The current direction in this field is to dissect how stromal cells recruit and regulate various immune suppressive cells to create an immunosuppressive TME. Single-cell RNA sequencing analysis provides a dynamic stromal niche that supports cancer stemness and immune evasion in various cancer types. Strikingly, this technique will provide mechanistic insight and a novel strategy for current immune checkpoint therapy. Traditionally, CSCs were regarded as subpopulations within the tumor that promote tumor recurrence and therapeutic resistance. Accumulating evidence has demonstrated the distinct role of CSCs in immune evasion. Immense effort has currently been made to dissect the crosstalk between CSCs and various immune suppressive cells, including MDSCs and Tregs. In addition, increasing interest is directed towards understanding the direct interaction between CSCs and macrophages and CD8^+^ T cells. Based on these interesting findings, CSC-targeted immunotherapy may be a promising approach for cancer therapy, and its therapeutic efficacy needs further investigation.

## Figures and Tables

**Table 1:** Therapeutic strategies targeting the interactions between CSCs and stromal cells for cancer treatments.

Type of Targeted Cell	Cancer Type	Novel Therapeutic Strategies	Mode of Action	Effects on Cancer Stemness	References
Targeted Stromal Cells	-				
Endothelial cells	Glioblastoma	IL-8 neutralizing antibody	Blockade of endothelial cell-secreted IL-8	Reduced spheroid size and tumor growth in vivo	[13]
Adipocytes	Breast/Mammary cancer	BMS309403	Inhibiting FABP4 functions	Suppressed tumor growth and tumor volume with decreased IL-6 level and tumor ALDH1 activity	[48]
	Ovarian cancer	Anti-OB-R blocking peptide	Blockade of leptin receptor	Decreased leptin-induced cell migration and invasion abilities	[51]
CAFs	Breast Cancer	GW4064	Agonist of FXR	Reduced progression and motility of tumors	[37,38]
		Pirfenidone (PFD) and doxorubicin	Inhibiting collagen production and tumor growth	Reduced components of ECM, inhibited tumor growth and lung metastasis	[39]
Targeted Immune Cells	-				
TAMs	Pancreatic carcinoma	CD40 Agonists	Activating and inducing macrophages	Degraded ECM and improved tumor infiltration of immune cells	[72]
	Non-small cell lung cancer (NSCLC)	IL-33 neutralizing antibody	Blockade of IL-33	Inhibited M2-like macrophages polarization via inhibition of IL-10 and VEGF as well as reduced accumulation of Treg cells	[73]
NK cells	Colon cancer	Chondrocytes	Expressing a high level of IL-12	Increased the infiltrations of both T cells and NK cells, reduced cancer cells and tumor angiogenesis	[91]
MDSCs	Breast cancer	Combination of anti-IL-6 antibody and iNOS inhibitor	Targeting MDSC-derived IL-6 and nitric oxide	Reduced spheroid formation stimulated by MDSCs	[97]
	Endometrial cancer	Doxorubicin and Gr-1 neutralizing antibody or celecoxib	Blockade of MDSCs or inhibiting MDSC functions	Reduced ALHD^+^ expression and sensitized tumor cells to chemotherapy	[105]
TANs	Breast cancer	Zileuton	Inhibiting neutrophil Alox5	Reduced lung metastasis	[115]
Treg cells	Glioma	Tocilizumab	Blockade of IL-6 receptor	Inhibited tumor growth and CD133 expression induced by Treg cells	[122]
	Acute myeloid leukemia (AML)	IL-10R neutralizing antibody	Neutralizing IL-10 receptor functions	Reduced side population, sphere formation ability, and expression of OCT4 and NANOG	[125]
	Ovarian cancer	CD25 neutralizing antibody	Inhibiting CD25^+^ Treg cells	Reduced angiogenesis and inhibited tumor growth	[127]

## Data Availability

No new data were created or analyzed in this study. Data sharing is not applicable to this article.

## Abbreviations

Protein kinase B, AKT; Aldehyde dehydrogenase, ALDH; Aldehyde dehydrogenase cytosolic 1, ALDH1; Aldehyde dehydrogenase 1 family member A1, ALDH1A1; Arachidonate 5-lipoxygenase, Alox5; acute myeloid leukemia, AML; axis inhibition protein 2, AXIN2; basic fibroblast growth factor, bFGF; cancer-associated fibroblasts, CAFs; chimeric antigen receptor-engineered natural killer, CAR-NK; C-C Motif Chemokine Ligand 1, CCL1; C-C motif chemokine ligand 17, CCL17; C-C Motif Chemokine Ligand 2, CCL2; Contaminant Candidate List 3, CCL3; prominin-1, CD133; FcγRIIIa, CD16; cluster of differentiation 24, CD24; IL-2 receptor α chain, CD25; sialic acid binding Ig-like lectin 3, CD33; cluster of differentiation 4, CD4; helper T cell, CD4+; Cluster of Differentiation 44, CD44; neural cell adhesion molecule, CD56; cytotoxic T cell, CD8+; Cluster Differentiation 90, CD90; cancer-initiating cells, CICs; tyrosine-protein kinase Met, c-met; cancer stem cells, CSCs; colony stimulating factor 1, CSF-1; C-terminal-binding protein 2, CtBP2; CXC chemokine ligand, CXCL; specific C-X-C motif chemokine receptor 2, CXCR2; DNA methyltransferase 3B, DNMT3B; decidual NK, dNK; extracellular matrix, ECM; epidermal growth factor, EGF; epithelial to mesenchymal transition, EMT; epithelial cell adhesion molecule, EpCAM; Epiregulin, EREG; extracellular signal-regulated kinase, ERK; Fatty acid-binding protein 4, FABP4; famegoid X receptor, FXR; forkhead box P3, FOXP3; Fos-related antigen 1, FRA1; granulin-epithelin precursor, GEP; hepatocellular Carcinoma, HCC; human epidermal growth factor receptor 2, HER2; Hes Related Family BHLH Transcription Factor With YRPW Motif 1, HEY1; Hepatocyte Growth Factor, HGF; insulin-like growth factor 1 receptor, IGF1R; Insulin-like growth factor 2, IGF-II; Interleukin IL; inducible NO synthase, iNOS; Janus Kinase/Signal Transducer and Activator of Transcription, JAK-STAT; Lymphoid Enhancer Binding Factor 1, LEF1; Mitogen activated protein kinases, lipoma-preferred partner, LPP; MAPK; myeloid-derived suppressor cells, MDSC; microRNA-101, miRNA101; Matrix metallopeptidase 9, MMP9; mechanistic target of rapamycin, mTOR; Myeloid differentiation factor 88, MYD88; Nanog Homeobox, NANOG; Natural cytotoxicity receptors, NCR; Nuclear factor kappa B, NF-κB; natural killer cells, NK; Natural killer group 2 member D, NKG2D; non-small-cell lung carcinoma, NSCLC; oligodendrocytes, O4; octamer-binding transcription factor 4, OCT4; Phosphoinositide 3-kinase, PI3K; Programmed death-ligand 1, PD-L1; Prostaglandin E2, PGE2; Procollagen-Lysine 2-Oxoglutarate 5-Dioxygenase 2, PLOD2; prostate cancer, PCa; Receptor tyrosine kinase, RTK; SRY-Box Transcription Factor 2, Sox2; Signal transducer and activator of transcription 3, STAT3; tumor-associated macrophages, TAMs; tumor-associated neutrophils, TANs; Transforming growth factor beta, tumor-associated NK; TANK, T helper 2 cell, Th2; Tissue inhibitor of metalloproteinase 1, tissue inhibitors of metalloproteinase; TIMP, Toll-like receptor 7; TLR7, tumor-infiltrating NK; TINK, tumor microenvironment, TME; tumor necrosis factor alpha, TNFα; regulatory T cells, Tregs; Vascular endothelial growth factor, VEGF; Vascular endothelial growth factor receptor 2, VEGFR2; Wingless-related integration site, Wnt.
